# A case of a resected mediastinal thymoma with spontaneous regression

**DOI:** 10.1016/j.ijscr.2025.112000

**Published:** 2025-10-02

**Authors:** Takahiko Hazemoto, Ryusei Yamada, Mayu Inomata, Ryo Maeda

**Affiliations:** Department of Thoracic and Breast Surgery, Faculty of Medicine, University of Miyazaki, Miyazaki, Japan

**Keywords:** Case report, Spontaneous regression, Thymoma, Video-assisted thoracic surgery

## Abstract

**Introduction and importance:**

We report a case of a resected thymoma with preoperative spontaneous regression in a 76-year-old woman. Only 13 cases of spontaneous regression of thymomas have been reported in the English literature, including this one.

**Case presentation:**

During a regular checkup, chest radiography revealed an abnormal shadow in the right hilum of an asymptomatic 76-year-old woman. Chest computed tomography (CT) revealed a 41 × 32 mm anterior mediastinal tumor. Six months later, she presented with sudden anterior chest pain. Chest CT revealed that the tumor had grown slightly to 43 × 42 mm. Chest CT performed one day preoperatively revealed that the tumor had rapidly shrunk in one month (to 26 × 23 mm) and contained areas of necrosis. Surgical resection was performed to obtain a definitive diagnosis. The postoperative diagnosis was a type AB thymoma, classified as pathological stage I (Masaoka's classification) with intratumoral necrosis.

**Clinical discussion:**

The spontaneous regression in the present case might have been related to the necrosis observed in the tumor. We postulate that vascular occlusion due to minute thromboembolism resulted in tumor necrosis. This might have caused inflammation around the tumor, thereby causing the patient's chest pain.

**Conclusion:**

Thymomas should be included in the differential diagnosis of mediastinal tumors with necrosis that spontaneously regress, and surgical resection is required despite such regression.

## Highlights

• We report on spontaneous regression of a thymoma
• Surgical resection is needed even if the tumor regresses spontaneously

## 1 Introduction

Reports of spontaneous cancer regression are rare and the underlying mechanisms remain elusive. This unusual event is estimated to occur in one of every 60,000–100,000 patients with malignant tumors [1].

Thymomas are malignant epithelial tumors, accounting for <0.5 % of all malignant tumors [2]. They usually have an indolent growth pattern, rendering them difficult to distinguish from benign growths [3]. Herein, we report a rare case of a resected thymoma that exhibited preoperative spontaneous regression, and we discuss its underlying mechanism. This study was conducted in accordance with the principles of the Declaration of Helsinki and the SCARE guidelines [4].

## 2 Case presentation

**Fig. 1:**
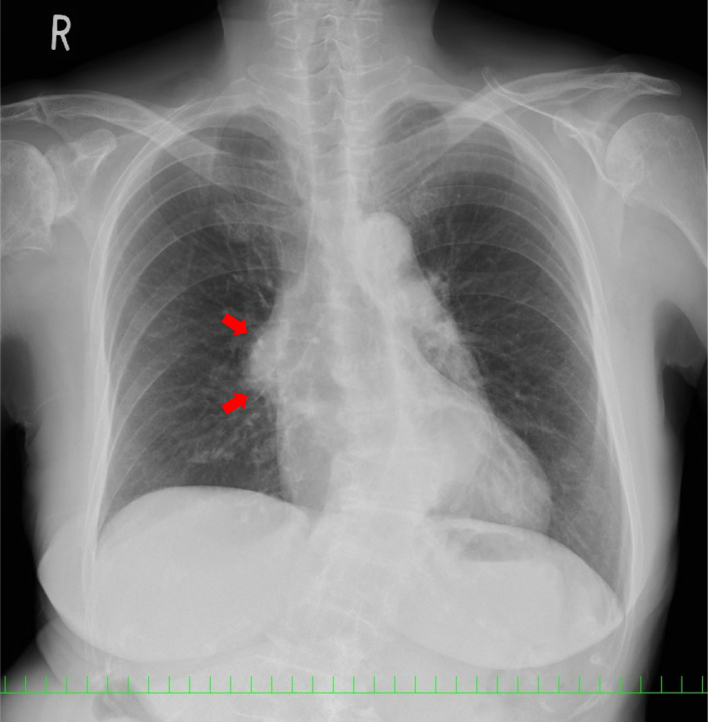
Chest radiograph showing a mass (red arrows) in the right hilum. (For interpretation of the references to colour in this figure legend, the reader is referred to the web version of this article.)

**Fig. 2:**
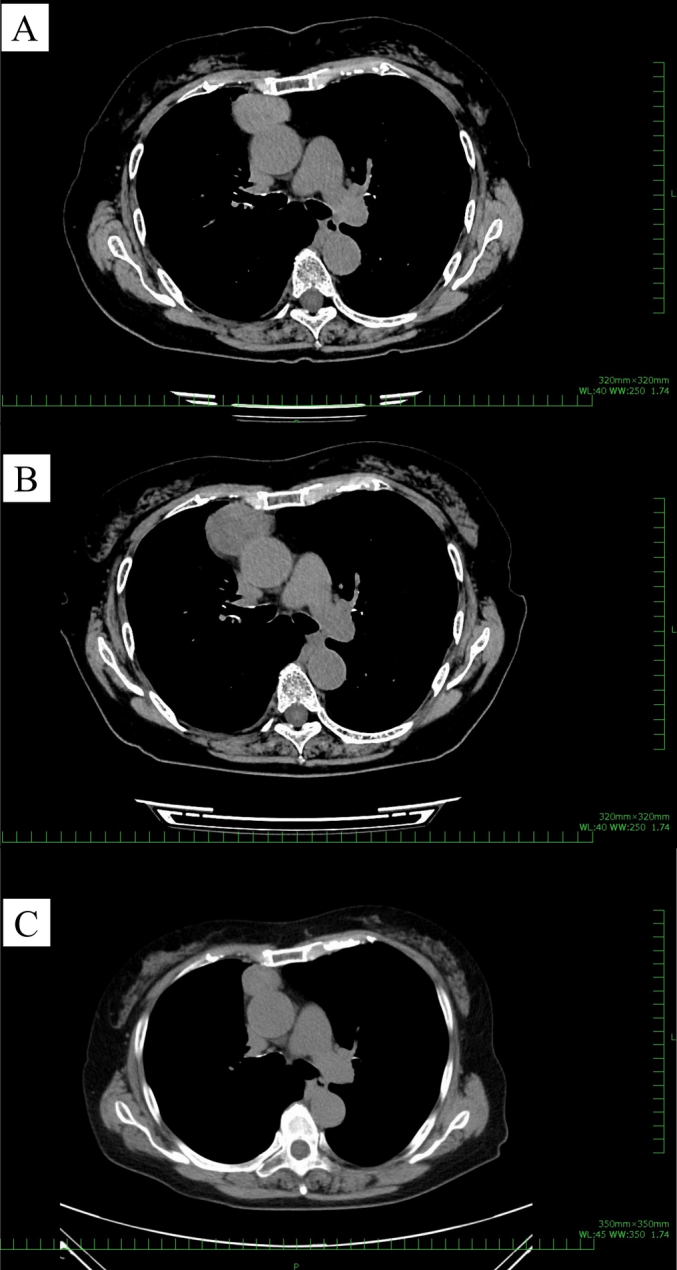
(A) Chest computed tomography (CT) showing a 41 × 32 mm anterior mediastinal tumor. (B) The anterior mediastinal tumor has slightly increased in size to 43 × 42 mm after 6 months. (C) Chest CT one day before the operation revealing that the tumor has rapidly shrunk in just one month, to 26 × 23 mm.

During a regular checkup, chest radiography revealed an abnormal shadow in the right hilum of an asymptomatic 76-year-old woman with no remarkable medical history or smoking history (Fig. 1). Chest computed tomography (CT) revealed a 41 × 32 mm anterior mediastinal tumor (Fig. 2A), but the patient declined further examination and treatment. Six months later, she presented with the chief complaint of sudden right anterior chest pain and low-grade fever that had persisted for 4 days. Chest CT revealed that the tumor had grown to 43 × 42 mm (Fig. 2B).

**Fig. 3:**
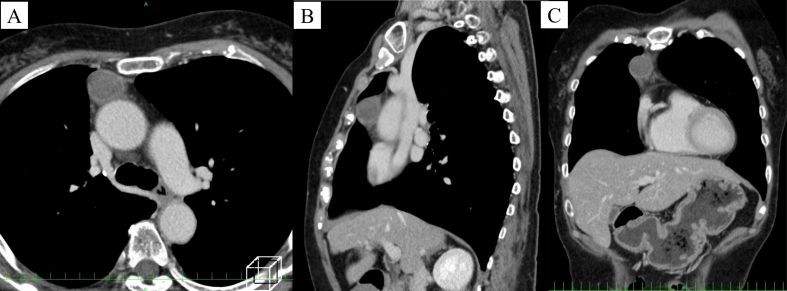
(A) Contrast-enhanced computed tomography showing a nodule with low attenuation in the center and enhancement at the periphery. (B) The sagittal view. (C) The coronal view.

The patient was referred to our hospital for further evaluation. Her tumor marker concentrations (carcinoembryonic antigen, cytokeratin fragment 21, progastrin-releasing peptide, α-fetoprotein, and human chorionic gonadotropin) were within the normal ranges. The patient's anti-acetylcholine receptor antibody concentration was normal. Because a malignant tumor was suspected and complete tumor resection was considered feasible, surgery was planned to obtain a definitive diagnosis and resect the tumor. However, chest CT one day before the operation revealed that the tumor had rapidly shrunk in just one month to 26 × 23 mm (Fig. 2C, Fig. 3). The patient's chest pain and fever had also been alleviated by that time.

Subxiphoid dual-port thymectomy was performed via video-assisted thoracic surgery with the patient in the supine position for a definitive diagnosis and complete resection. A 2.5-cm skin incision was made approximately 1 cm caudal to the xiphoid process. The additional 5-mm port was placed in the right lateral thoracic region. The operative time was 31 min, and the patient lost <10 mL blood. The chest tube was removed postoperatively on the day of surgery. The patient recovered without event and was discharged on the third postoperative day.

**Fig. 4:**
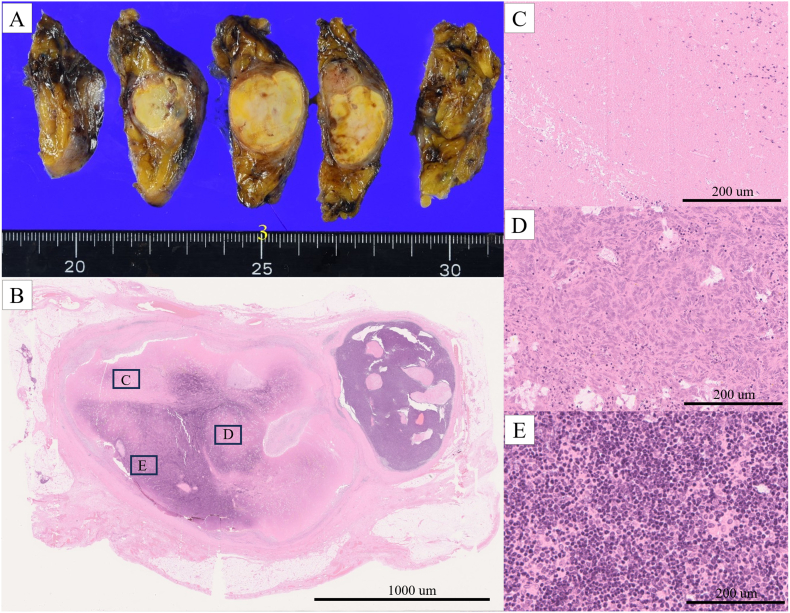
Pathology findings. (A) The tumor is encapsulated, with a white cut surface and well-circumscribed, yellowish-white internal area. (B) The tumor contains a broad necrotic area (hematoxylin–eosin staining). (C) A high-power view of a necrotic area (hematoxylin–eosin staining). (D) Histopathological examination showing a type AB thymoma, with a component of type A thymoma with short spindle cells (hematoxylin–eosin staining). (E) A component of type B thymoma with abundant infiltration of T lymphocytes (hematoxylin–eosin staining).

The tumor was encapsulated, with a white cut surface and well-circumscribed, yellowish-white internal area (Fig. 4A). Postoperative histological examination revealed a type AB thymoma (stage I according to the Masaoka classification). The tumor had large necrotic areas (Fig. 4C). No vessel occlusion was observed upon histopathological examination. No tumor cells were observed at the margins of the resected specimen.

The patient was examined at 3-month intervals for the first 24 months. The follow-up evaluation included physical examination and chest radiography. Chest CT was performed at 6-month intervals. She had no signs of recurrence and remained symptom-free for 24 months after surgery.

## 3 Discussion

Spontaneous tumor regression was first reported by Cole and Everson in 1956 and is defined as the partial or complete involution of a malignant tumor without the application of a specific therapy [5]. As mentioned in the introduction, it is exceedingly rare [1].

Thymomas originate from thymic epithelial cells. Among the rare tumors of the anterior mediastinum, thymomas are the most common primary tumors, occurring in approximately one per million individuals per year [6,7]. Although all thymomas are malignant, they are often difficult to discern from benign growths owing to their typically indolent growth pattern [3].

Spontaneous tumor regression is reportedly more frequent among certain malignancies, such as renal cell carcinoma, neuroblastoma, and malignant melanoma [1], whereas it is relatively rare among thymomas, with only 13 cases, including this one, reported in the English literature (Table 1) [[8], [9], [10], [11], [12], [13], [14], [15], [16], [17]].

**Table 1:** Reported cases of thymoma with spontaneous regression.

				Symptom	Tumor size on chest CT (mm)		Reduction rate (%)	Duration of SR (weeks)	Pathological findings		Masaoka stage	Follow-up (months)	The cause of SR proposed owing to the authors
Case	Author	Age	Sex	Chest pain	Before SR	After SR			Necrosis	WHO type			
1	Okagawa et al. [8]	31	F	+	60 × 55	45 × 40	45	3	+	B2	II	ND	Ischemic infarction by the rapid tumor growth
2	Hori et al. [9]	38	M	+	28	15		4	+	B2	II	9, alive without disease	Thromboembolism
3	Yutaka et al. [10]	47	M	+	80 × 70	60 × 30	68	4	+	B3	III	12, alive without disease	Ischemic infarction by the rapid tumor growth
4	Nakazono et al. [11]	49	F	−	ND	ND		2	+	B2	I	ND	ND
5	Nakazono et al. [11]	46	F	+	ND	ND		24	+	ND	I	ND	ND
6	Huang et al. [12]	52	F	−	25 × 20	0	100	36	−	B2	ND	12, alive without disease	Unknown
7	Fukui et al. [13]	43	F	+	34 × 34	14 × 12	85	6	+	B2	II	ND	Ischemic infarction by the rapid tumor growth
8	Fukui et al. [13]	32	F	+	100 × 95	73 × 69	89	1	+	B2	IVa	ND	Ischemic infarction by the rapid tumor growth
9	Toyokawa et al. [14]	28	M	+	118 × 58	100 × 38	44	3.5	−	B2	II	5, alive without disease	Immunological factors
10	Furuya et al. [15]	30	M	+	110 × 60	80 × 36	56	4	+	B2	II	12, alive without disease	Ischemic infarction by the rapid tumor growth
11	Kikuchi et al. [16]	71	M	−	32 × 31	23 × 15	65	8	−	B3	I	12, alive without disease	Unknown
12	Nishina et al. [17]	44	M	+	55 × 43	30 × 30	62	192	+	B2	II	6, alive without disease	Ischemic infarction by the rapid tumor growth
13	Present case	76	F	+	43 × 42	26 × 23	67	4	+	AB	I	24, alive without disease	Thromboembolism

CT; computed tomography, SR; spontaneous regression, WHO; World Health Organization, F; female, M; male, ND; not described, +; positive, −; negative.

More than one-third of patients with thymomas present with systemic syndromes, including myasthenia gravis and pure red cell aplasia [3]. Thymomas have been observed in 15 % of patients with myasthenia gravis and 50 % of those with pure red cell aplasia [3]. Oral glucocorticoids for these systemic syndromes may induce radical thymoma regression [18]; however, our patient did not take any medication during the regression.

Although the underlying mechanism of the spontaneous regression of malignant tumors is difficult to determine scientifically and definitively, immune mechanisms are considered to play a central role in the observed regression of renal cell carcinoma and malignant melanoma [19,20]. Other possible mechanisms that affect spontaneous regression include tumor necrosis [21], cytokine changes [22], apoptosis [23], psychological factors [24], genetic and epigenetic factors [25], and the induction of benign differentiation [26]. In the present study, preoperative CT revealed a peripherally enhanced mass with some areas of necrosis within the tumor. Histological analysis confirmed that the tumor had a large necrotic area. The phenomenon of spontaneous regression observed in the present case may be related to this necrosis. According to Nishina et al., one possible cause of tumor necrosis of a thymoma is rapid enlargement prior to regression, leading to disruption of the vascular supply and necrosis [17]. However, in the present case, CT revealed no such rapid increase in size in the 6 months prior to shrinkage.

Thymomas are generally asymptomatic, and the majority of patients commonly present with abnormal chest shadows [2]. In contrast, in the present case, the patient experienced sudden chest pain before the tumor decreased in size. We hypothesize that vascular occlusion due to minute thromboembolism resulted in tumor necrosis, although no thromboembolism was detected pathologically. A thromboembolism might have caused inflammation around the tumor, thereby causing the patient's chest pain. In previous reports, patients with spontaneous regression of thymomas also presented with symptoms such as fever or chest pain [8,15]. However, the cause, mechanism, and trigger of regression, as well as the cause of necrosis, have not been clearly determined and remain a hypothesis.

Surgical excision is the gold standard treatment for resectable thymomas [2]. Clinicians should recognize the possibility of spontaneous regression among patients with thymomas and that surgical resection is needed even if an anterior mediastinal tumor regresses spontaneously.

## 4 Conclusion

We reported the case of a patient with a thymoma that spontaneously regressed. The differential diagnosis of mediastinal tumors with necrosis that spontaneously regress should include thymomas, and surgical resection is required despite spontaneous regression.

## Abbreviations

CT: computed tomography

## Consent

Written informed consent was obtained from the patient for publication of this case report and accompanying images. A copy of the written consent is available for review by the Editor-in-Chief of this journal upon request.

## Ethics approval

As it is a case report, ethical approval is exempted by University of Miyazaki Hospital.

## Funding

The authors have no competing interests to declare.

## Author contribution

Dr. Ryo Maeda is the writer of this article and corresponding author.

Dr. Takahiko Hazemoto, Dr. Ryusei Yamada, and Dr. Mayu Inomata have reviewed.

## Guarantor

Dr. Ryo Maeda accepts all responsibility of this article.

## Research registration number

Not applicable.

## Conflict of interest statement

The authors have no competing interests to declare. All authors have read and approved the final manuscript.
